# Physiological and Pathological Roles of Mammalian NEK7

**DOI:** 10.3389/fphys.2020.606996

**Published:** 2020-12-07

**Authors:** Zhenzhen Sun, Wei Gong, Yue Zhang, Zhanjun Jia

**Affiliations:** ^1^Department of Nephrology, Children’s Hospital of Nanjing Medical University, Nanjing, China; ^2^Nanjing Key Laboratory of Pediatrics, Children’s Hospital of Nanjing Medical University, Nanjing, China; ^3^Jiangsu Key Laboratory of Pediatrics, Nanjing Medical University, Nanjing, China

**Keywords:** NEK7, mitosis, cellular homeostasis, NLRP3 inflammasome, inhibitors

## Abstract

NEK7 is the smallest NIMA-related kinase (NEK) in mammals. The pathological and physiological roles of NEK7 have been widely reported in many studies. To date, the major function of NEK7 has been well documented in mitosis and NLRP3 inflammasome activation, but the detailed mechanisms of its regulation remain unclear. This review summarizes current advances in NEK7 research involving mitotic regulation, NLRP3 inflammasome activation, related diseases and potential inhibitors, which may provide new insights into the understanding and therapy of the diseases associated with NEK7, as well as the subsequent studies in the future.

## Introduction

Mammalian NIMA-related kinases (NEKs) represent a family of serine/threonine kinases, named NEK1–NEK11, which are implicated in the control of several aspects of mitosis and are involved in non-mitotic functions (Forrest et al., 2003; O’Regan et al., 2007; Fry et al., 2012; Cullati et al., 2017). Members of the NEK family are conserved proteins in structure, sharing approximately 40–45% identity with NIMA within their C-terminal catalytic kinase domains (Kandli et al., 2000). Of these NEK family member, NEK7 is the smallest protein, composed of only a catalytic domain with a 30–40 amino acid N-terminal extension, which shares more than 85% sequence identity to NEK6 (Kandli et al., 2000; Kimura and Okano, 2001). Nevertheless, NEK7 and NEK6 show divergent cellular functions as a result of the differential spatiotemporal tissue distribution and enzymatic control (Belham et al., 2001; Kimura and Okano, 2001; Feige and Motro, 2002; Minoguchi et al., 2003; de Souza et al., 2014). Further analysis led to the discovery that *NEK7* gene is on chromosome 1 and that its ORF encodes a 302-amino-acid polypeptide with a molecular mass of 34.5 kD (Kimura and Okano, 2001; Katoh and Katoh, 2004). NEK7 is widely expressed in various tissues, such as the heart, liver, lung, brain, muscle, testis, leukocyte, and spleen (Kimura and Okano, 2001). Accumulating evidence suggests that NEK7 is involved in mitosis regulation through an intricate mechanism (Belham et al., 2003; Forrest et al., 2003; Quarmby and Mahjoub, 2005; Fry et al., 2012).

The NLRP3 inflammasome is an intracellular multiprotein complex that assembles NLRP3, ASC, and pro-caspase-1, which leads to the activation of caspase-1, the cleavage and secretion of interleukin 1β (IL-1β) and interleukin 18 (IL-18) in response to diverse stimuli (He et al., 2016a; Mathur et al., 2017). With the inappropriate release of proinflammatory cytokines, the NLRP3 inflammasome is involved in various inflammatory diseases, such as atherosclerosis, type 2 diabetes, Alzheimer’s disease, gout, rheumatoid arthritis, and inflammatory bowel disease (Liu D. et al., 2020). In recent years, NEK7 has been demonstrated to be essential for canonical NLRP3 inflammasome activation by directly binding to the leucine-rich repeat (LRR) domain of NLRP3 (Shi et al., 2016; Sharif et al., 2019). Furthermore, increasing evidence suggests the important role of NEK7 in the development of NLRP3 inflammasome-related diseases (Xu et al., 2016; Nozaki and Miao, 2019). Therefore, the documented pathogenesis mechanism of NLRP3 inflammasome activation by NEK7 strongly indicates promising roles for targeting NEK7 in treating inflammation-related diseases (Xu et al., 2016).

Clearly, cell division and NLRP3 inflammasome activation are extremely important to normal cellular process and stress responses of organisms. NEK7 drew attention two decades ago, but its function has not been explored completely. Several reviews have summarized the partial role of NEK7 by focusing on single aspects of its function, especially NLRP3 inflammasome activation (Fry et al., 2012; He et al., 2016a; Xu et al., 2016; Liu G. et al., 2020). An extensive overview and detailed classification of the unique molecular mechanisms of NEK7, highlighting its potential as therapeutic targets, may provide novel insight into preventing and treating a host of related diseases.

## NEK7 in the Regulation of Mitosis

To date, the role of NEK7 in mitotic progression has been the best characterized function, including centrosome enrichment and microtubule nucleation, which are essential for centriole duplication and centrosomal pericentriolar material (PCM) protein accumulation during interphase, centrosome separation in prophase and proper spindle assembly in metaphase (Yissachar et al., 2006; Kim et al., 2007; O’Regan and Fry, 2009; Salem et al., 2010; Sdelci et al., 2011). Nevertheless, NEKL-3 in *Caenorhabditis elegans*, although highly homologous to mammalian NEK6/NEK7, showed only molting functions, including the regulation of the apical extracellular matrix, intracellular trafficking and endocytosis, but not its role in mitosis (Yochem et al., 2015; Lazetic and Fay, 2017; Lazetic et al., 2018; Liu D. et al., 2020).

Emerging evidence indicates the possible involvement of NEK7 in centrosome formation and separation, microtube nucleation and spindle assembly (Yissachar et al., 2006; Kim et al., 2007; O’Regan and Fry, 2009; Salem et al., 2010; Sdelci et al., 2011). Endogenous NEK7 protein was initially shown to be enriched at the centrosome throughout all phases of the cell cycle (Kim et al., 2007). Additional studies revealed that the NEK7 signal was also detected temporally in the midbody, spindle poles and cytoplasm, in addition to the centrosome (Yissachar et al., 2006). Established works have demonstrated in detail that NEK7 is essential for centriole duplication and centrosomal accumulation of pericentriolar material proteins in interphase cells (Kim et al., 2011). Importantly, NEK7 deficiency results in a significant increase in the number of mitotic cells acquiring a multipolar or monopolar spindle phenotype and ultimately leads to cell arrest at G1 phase, prometaphase, metaphase, and cytokinesis of anaphase in the cell cycle (Kim et al., 2007). In addition, a decrease in centrosomal γ-tubulin levels and microtubule nucleation activity was observed in NEK7-suppressed cells (Kim et al., 2007; Cohen et al., 2013). Thus, microtubule regulation was thought to be the intermediate mechanism through which NEK7 regulated mitosis progression.

Microtubules are composed of highly dynamic filaments that connect kinetochores to the mitotic spindle poles critical for aligning and segregating chromosomes (Cohen et al., 2013). This precise network has long been suspected to be a major target of NEKs. NEK7 was initially found to be related to centrosomal γ-tubulin levels in mitotic cells in an earlier study (Kim et al., 2007). How γ-tubulin is regulated by NEK7 remains unclear. Another study provided a preliminary hint that Nercc1/NEK9 catalyzed the direct phosphorylation of prokaryotic recombinant NEK6 at Ser^206^ and probably participated in the regulation of NEK7 in a similar manner (Belham et al., 2003). Conformation and activity analyses showed that the C-terminal domain of NEK9 interacts with NEK6/NEK7 through the release of Tyr97 autoinhibition (Richards et al., 2009), a finding confirmed by a later study (Haq et al., 2015). However, the evidence that this function has an effect on mitotic progression is limited.

Several studies have found that Plk1 was likely the indispensable upstream activator of the NEK9/NEK6/NEK7 cascade in controlling early centrosome separation (Bertran et al., 2011; Sdelci et al., 2011, 2012; Dodson et al., 2013). Notably, one study found that NEK9 phosphorylation by CDK1 and Plk1 ultimately resulted in the phosphorylation of NEK6/NEK7 and the mitotic kinesin Eg5, which are necessary for normal centrosome separation during prophase (Bertran et al., 2011). In another study, DYNLL/LC8 increased the autophosphorylation of Ser944 of NEK9 by binding to a (K/R)XTQT motif adjacent to the NEK9 C-terminal coiled-coil motif, which directly interferes with NEK9 binding to its downstream partner NEK6/7 (Regue et al., 2011). This observation has been confirmed by a structural analysis in a later study by the same authors (Gallego et al., 2013). In addition, one study using Xenopus egg extracts and mammalian cells showed that NEK9 phosphorylated NEDD1 on Ser377, driving its recruitment of γ-tubulin to the centrosome by Plk1-dependent phosphorylation in mitotic cells (Sdelci et al., 2012). However, this study did not find evidence for a role of NEK7 in centriole duplication, in contrast to previous studies (Kim et al., 2011). However, in a recent study, a detailed investigation still showed the important impact of NEK7 depletion on contributions to centrosomal accumulation of the APC/C cofactor Cdh1, which negatively regulates centriole duplication (Gupta et al., 2017). Later, RGS2 was reported to be a novel interactor of NEK7. NEK7 binds to and phosphorylates RGS2 and leads to the localization of RGS2 to the mitotic spindle, which is required for proper mitotic spindle organization and spindle orientation (de Souza et al., 2015). In addition, NEK6 and NEK7 promote the dissociation of EML4 from microtubules in mitosis by phosphorylating the EML4 N-terminal domain at Ser144 and Ser146, which is required for efficient chromosome congression in interphase (Adib et al., 2019). As a potential function of NEK7 in microtube regulation (Cohen et al., 2013), NEK7 was identified to regulate dendrite growth and branching, as well as spine formation and morphology, in part through phosphorylation of the kinesin Eg5/KIF11 (Freixo et al., 2018). At approximately the same time, another study also found that NEK7 specifically controlled the shape and synaptic outputs of cortical parvalbumin interneurons (Hinojosa et al., 2018). However, these studies did not show the cell division-related role of NEK7. Summing up the above studies, the exact role of NEK7 in mitosis regulation needs to be further investigated.

## NEK7 in the Regulation of NLRP3 Inflammasome Activation

**FIGURE 1:**
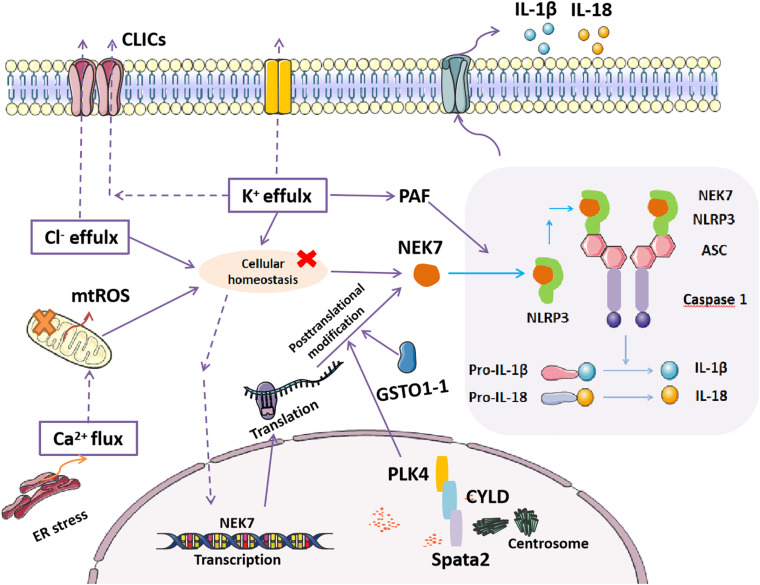
Mechanisms of NEK7-mediated NLRP3 inflammasome activation. NEK7 directly binds with NLRP3, which triggers canonical NLRP3 inflammasome activation, including the activation of caspase 1, cleavage of pro-IL-1β, and pro-IL-18, producing mature IL-1β and IL-18, which are secreted to the extracellular space as inflammatory effectors. Disordered cellular homeostasis, including K^+^ efflux, Ca^2+^ signaling, chloride efflux, and mitochondrial dysfunction (mtROS), are the upstream signals that regulate NEK7-mediated NLRP3 inflammasome activation by either upregulating NEK7 expression or inducing the binding of NEK7 to NLRP3. Post-translational modification of NEK7 regulated by centrosome proteins (spata, CYLD, and PLK4) and the deglutathionylating enzyme GSTO1-1 that inhibits or induces the binding of NEK7 to NLRP3, respectively, affect NLRP3 inflammasome activation.

Generally, the activation of the NLRP3 inflammasome requires the synergistic effect of two signals (He et al., 2016a). Signal 1 (priming), such as the microbial component LPS and other endogenous cytokines, promotes the upregulation of inflammasome components as well as its newly discovered member, NEK7, as the protein levels of NLRP3 and NEK7 in resting cells are thought to be insufficient for NLRP3 activation (He et al., 2016a; Shi et al., 2016). Recent reports also showed that post-transcriptional regulation and post-translational modifications of NEK7 and NLRP3 were essential for inflammasome assembly (Song et al., 2017; Hughes et al., 2019; Gritsenko et al., 2020). Signal 2 (activation), such as ATP, pore-forming toxins, viral RNA, and particulate matter, accelerates the activation of the NLRP3 inflammasome, resulting in the activation of caspase 1 and the maturation and secretion of IL-1β and IL-18 (He et al., 2016a). In recent years, in addition to its mitotic function, NEK7 has been better understood for its direct binding with NLRP3, which is indispensable for NLRP3 inflammasome activation (He et al., 2016b; Shi et al., 2016; Nozaki and Miao, 2019; Figure 1).

Initially, through a forward genetic analysis, mice with a mutation in NEK7 were found to display diminished IL-1β secretion, and further evidence revealed that NEK7 binds directly to the leucine-rich repeat (LRR) domain of NLRP3 downstream of the induction of mitochondrial reactive oxygen species (ROS) (Shi et al., 2016). The interaction promotes the assembly and activation of the NLRP3 inflammasome, for the first time suggesting that NEK7 is a component of the NLRP3 inflammasome (Shi et al., 2016). Intriguingly, this study also showed that NEK7 cannot be available for inflammasome activation and mitosis simultaneously because of the limiting amount of it in cells (Shi et al., 2016). Later, compelling cumulative evidence confirms the direct binding of NEK7 and NLRP3, and their interaction in inflammasome activation is regulated under diverse conditions (Gross et al., 2016; Hoss et al., 2019; Nozaki and Miao, 2019; Sharif et al., 2019). Intriguingly, a recent study found that NEK7 was dispensable for NLRP3 inflammasome formation in human and murine cells under pro-inflammatory conditions (Schmacke et al., 2019).

The NLRP3 inflammasome is activated by diverse stimuli, including multiple microbial products, endogenous molecules, and particulate matter (Shi et al., 2016; Mathur et al., 2017; Liu D. et al., 2020). Furthermore, an increasing number of studies have demonstrated that disordered cellular homeostasis following these stimuli is the proximal upstream trigger of NLRP3 inflammasome activation (Shi et al., 2016). Cellular homeostasis are disrupted by various stimuli, including K^+^ efflux, Ca^2+^ signaling, chloride efflux, mitochondrial dysfunction, ROS, and lysosomal rupture, which are indispensable for the downstream interaction of NEK7 and NLRP3 (He et al., 2016b; Shi et al., 2016; Tang et al., 2017; Green et al., 2018). The phospholipid platelet-activating factor (PAF) activates the NLRP3 inflammasome in the presence of NEK7 in a mechanism that depends on calcium and potassium flux but not on ROS or cathepsin (Deng et al., 2019). However, as shown in an earlier study, cytochrome c released from stressed or damaged mitochondria negatively regulates NLRP3 inflammasome activation by competitively binding to the LRR domain of NLRP3, thus reducing the interaction between NLRP3 and NEK7 (Akiyama et al., 2016).

In addition, emerging studies have revealed that the transcriptional and post- transcriptional regulation of NEK7 and NLRP3 are also critical for the activation of the NLRP3 inflammasome. NF-kB has been previously reported to upregulate the expression of components of the NLRP3 inflammasome. Recently, a study found that RELA, a subunit of NF-kB, can also transcriptionally upregulate the expression of NEK7, thereby leading to the subsequent interaction of NEK7 and NLRP3 (Chen X. et al., 2019). Alternative splicing (AS) of pre-mRNA determines the generation of various proteins from individual gene with distinct functions. Evidence has indicated stochastic AS of the LRR domain in human *NLRP*3, but not mouse *NLRP3*, including the full-length variant and a variant lacking exon 5; the protein encoded by the latter variant cannot interact with NEK7 (Hoss et al., 2019). This finding suggests that LRR in NLRP3 that interacts with NEK7 is encoded by exon 5 (Hoss et al., 2019). At the time of this discovery, another study showed that the endogenous deglutathionylating enzyme GSTO1-1 is involved in the activation of the NLRP3 inflammasome through the mechanistic deglutathionylation of NEK7 of cysteine 253 (Hughes et al., 2019). However, a subsequent study found a novel complicated post-translation modification of NEK7. Specific centrosome-localized spata2 recruits CYLD for the deubiquitination of polo-like kinase 4 (PLK4), which further binds to and phosphorylates NEK7 at Ser204 (Yang et al., 2019). Thereby, this phosphorylation modification of NEK7 suppressed its binding with NLRP3 and inhibited NLRP3 inflammasome activation (Yang et al., 2019). In addition, HDAC6-mediated MTOC localization of NLRP3 may ensure the engagement of centrosomal kinase NEK7 (Magupalli et al., 2020). These studies suggest the important role of NEK7 in NLRP3 inflammasome activation. However, the mechanism by which NEK7 switches from a cell cycle regulator to an inflammasome regulator remains unclear (Yang et al., 2019). In conclusion, it is imperative to characterize the precise regulation of NEK7 involved in NLRP3 inflammasome activation for finding more refined therapeutic targets for related diseases.

## Other Functions of NEK7

Other functions of NEK7, except for cell division and the NLRP3 inflammasome activation, have been uncovered in recent years. During the exploration of the protein kinases related to the formation of hippocampal long-term potentiation (LTP), NEK7 was found to be distributed in hippocampal areas and downregulated upon the induction of LTP (Li et al., 2014). In another similar study, NEK7 was found to be a candidate for the neurotransmitter system and immunomodulation by genome-wide association analysis (Gley et al., 2019). However, these studies are observational and the mechanisms remains obscure. In addition, the importance of NEK7 in maintaining telomere integrity has been studied (Tan et al., 2017). In response to damage, NEK7 is recruited to telomeres and directly phosphorylates TRF1 on Ser114, which prevents the subsequent proteasomal degradation (Tan et al., 2017). These studies have implied that NEK7 may be involved in other cellular processes in addition to the regulation of mitosis and the NLRP3 inflammasome. Therefore, further studies are needed to comprehensively understand the role of NEK7.

## NEK7-Regulated Mitosis and NLRP3 Inflammasome in Diseases

### Diseases Related to Mitosis

Established evidence shows that NEK7 is involved in regulating various aspects of microtubule stability, spindle formation and cytokinesis. Atypical expression or post-translation modification of NEK7 leads to spindle disorganization, cytokinesis disturbance, micronuclei formation, mitotic arrest, and even cell death. An early study reported that the absence of *NEK7* leads to lethality in late embryogenesis or at early postnatal stages and to severe growth retardation in mice (Filonenko et al., 2007; Salem et al., 2010). Furthermore, the intimate connection between microtubule instability and unregulated cell division and cancer development suggests that NEK7 has a potential role in oncogenesis. Accordingly, in the last decade, a host of studies have demonstrated the potential role of NEK7 in the cancer development of various tissues. This evidence suggests that NEK7 is a potential therapeutic target for diseases related to mitotic regulation.

Initially, NEK7 was thought to be the downstream target of WHSC1L1 involved in human carcinogenesis through expression profile analysis *in vitro* using human bladder and lung cancer cell lines (Kang et al., 2013). Similarly, in a recent study, EML4-ALK variant 3(V3) was proposed to mediate microtubule stabilization through NEK7 and NEK9, accelerating cell migration in EML4-ALK lung cancer (O’Regan et al., 2020). Another study found *NEK7* is probably a downstream target gene of WHSC1 in multiple squamous cell carcinoma of the head and neck (SCCHN) cell lines as indicated via microarray expression profile analysis (Saloura et al., 2015). Chromatin immunoprecipitation (ChIP) assays showed *NEK7* was directly regulated by WHSC1 through H3K36me2 (Saloura et al., 2015). However, solid evidence is limited. Notably, in gallbladder cancer, a significant correlation was first observed between the increased protein expression of NEK7, FoxM1 and Plk1 and tumor differentiation, development and shorter overall survival time (Wang et al., 2013). However, how NEK7 is involved in gallbladder cancer needs to be further determined. Similarly, high NEK7 expression was also significantly correlated with hepatocellular carcinoma (HCC), with the degree of malignancy, as reflected in tumor numbers, tumor diameter, adjacent organ invasion, tumor grade, and TNM stage (Zhou et al., 2016). The downstream target was preliminarily focused on cyclin B1, as silencing of *NEK7* resulted in decreased cyclin B1 levels both *in vitro* and *in vivo* (Zhou et al., 2016). Nevertheless, a later study demonstrated that silencing *NEK7* resulted in reduced CDK2, cyclin D1, and cyclin E levels *in vitro*, which therefore significantly inhibited retinoblastoma cell (Y79, SO-RB50 and WERI-RB1) proliferation (Zhang J. et al., 2017). The role of NEK7 in prompting retinoblastoma progression was first discovered in a refined meta-analysis of retinoblastoma copy numbers (Krahe et al., 2016). However, the detailed mechanism is still unclear. A recent breast cancer-related study provides solid evidence that UNC45A nuclear localization promotes the expression of the mitotic kinase NEK7 and that the mitotic catastrophe resulting from UNC45A deficiency can be rescued by heterologous NEK7 expression (Eisa et al., 2019). Furthermore, detailed work including computational sequence analysis, RNA-seq data, ChIP-qPCR, and EMSAs have indicated that two novel glucocorticoid response elements (GREs) exist upstream of the *NEK7* transcription start site (TSS) and that the glucocorticoid receptor (GR) is a positive regulator downstream of UNC45A that promotes *NEK7* gene transcription (Eisa et al., 2019). However, these findings are based only on experiments performed *in vitro*, and *in vivo* studies are necessary to ultimately confirm the mechanisms (Eisa et al., 2019). Additionally, in a previous study, *Anks3*, an interactor of nephronophthisis (NPH, an autosomal recessive cystic kidney disease)-related gene *Anks6*, may contribute to the nuclear exclusion of NEK7 and prevent undesired re-entry of interphase cells into the cell cycle (Ramachandran et al., 2015). This is the only study of NEK7 in mitosis regulation related to kidney disease (Ramachandran et al., 2015). However, the detailed mechanism and spatiotemporal relationship remain unclear. Comprehensively summarizing the current knowledge of NEK7 function in various aspects of cell division and related diseases will highlight potential targets for effective therapeutic strategies.

### Diseases Related to the NLRP3 Inflammasome

In recent years, NEK7-mediated NLRP3 inflammasome activation has been reported to be involved in various inflammatory diseases (Xu et al., 2016; Chen X. et al., 2019; Gomes Torres et al., 2019). A significant correlation was shown between gene polymorphisms in *NEK7*, *TLR* (toll-like receptors), *NLR* (nod-like receptors), and lipid and glucose parameters from healthy children and adolescents and adults (Gomes Torres et al., 2019). This finding suggests a role of NEK7 in metabolic and inflammatory disorders. A later study showed that NEK7 may also be involved in inflammatory bowel disease mediated by NLRP3 inflammasome activation, the mechanism of which has been described in the previous section (Chen X. et al., 2019). Similarly, several studies demonstrated that the development of neuroinflammation post-traumatic brain injury, ventilator-induced lung injury, diabetic periodontitis, DSS-induced ulcerative colitis, and endometritis in cattle display a significant correlation with NEK7-involved NLRP3 inflammasome activation (Chen Y. et al., 2019; Kelly et al., 2019; Liu et al., 2019; Liu H. et al., 2020; Zhou et al., 2019, 2020; Cao et al., 2020). In a previous study, *NEK7* was predicted to be a target for miR-664 involved in the proinflammatory response to influenza A (H7N9) virus, but direct evidence was lacking (Chan et al., 2016). In contrast, clinical analysis in patients with systemic lupus erythaematosus (SLE) revealed a negative correlation between the levels of NEK7, NLPR3, and ASC and disease development, whereas a positive correlation was observed with IL-1β and IL-18 (Ma et al., 2018). This finding suggests that the NEK7-NLRP3 complex might play a protective role (Ma et al., 2018). However, the detailed mechanism remains unclear. As a mediator of NLRP3 inflammasome assembly and activation, NEK7 may also be associated with other related diseases resulting from the improper activation of the NLRP3 inflammasome, which requires further evidence.

## Inhibitors and Their Application to Diseases

As a critical component of the NLRP3 inflammasome, NEK7 contributes to various pathologies of NLRP3 inflammasome-related diseases (Xu et al., 2016). Some promising selective inhibitors and medicines already used to treat other diseases are found to target various aspects of inflammasome activation regulated by NEK7. These inhibitors may disrupt the upstream events or directly regulate the expression or modification of NEK7 and NLRP3 thus effecting their interaction (Zhang Y. et al., 2017; He et al., 2018; Chiu et al., 2019; Wu et al., 2019; Liu D. et al., 2020; Shi et al., 2020).

Among these inhibitors, MCC950 is the best characterized for its potent effect in many diseases with NEK7-NLRP3-mediated inflammasome activation (Wu et al., 2019), including high glucose-induced human retinal endothelial cell dysfunction (diabetic retinopathy) (Zhang Y. et al., 2017), lung ischemia-reperfusion injury (Xu et al., 2018), endometritis in cattle and peritonitis (Kelly et al., 2019). Although some studies are limited to *in vitro* applications, MCC950 is still considered to be a promising treatment, as it selectively blocks the interaction between NEK7 and NLRP3 (Wu et al., 2019). In addition, several inhibitors target genes that have been found to be involved in the regulation of NEK7-mediated NLRP3 inflammasome activation, such as C1-27 (GSTO1-1 inhibitor) (Hughes et al., 2019), IAA94 (CLIC inhibitor) (Tang et al., 2017), and JSH-23 (p65 inhibitor) (Chen X. et al., 2019). These inhibitors display a prominent attenuating effect on NEK7-related NLRP3 inflammasome activation, as indicated by *in vitro* and *in vivo* experiments under certain conditions.

Importantly, a growing number of studies have suggested that some drugs, including natural products (traditional Chinese medicine) (He et al., 2018; Kim et al., 2019; Shi et al., 2020) and chemical medicines (Zhang et al., 2018; Chiu et al., 2019; Torp et al., 2019; Zhou et al., 2019), have emerging roles in inhibiting NEK7-related NLRP3 inflammasome activation with distinct regulatory mechanisms. The three natural products oridonin (He et al., 2018; Liu H. et al., 2020), artemisinin (Kim et al., 2019), and ginsenoside Rg3 (Shi et al., 2020) have been reported to have similar functions in abrogating NEK7-NLRP3 interaction, as corroborated by different mouse models (He et al., 2018; Kim et al., 2019; Shi et al., 2020). Seven chemical medicines, including glucosamine (Chiu et al., 2019), metformin (Zhou et al., 2019, 2020), glibenclamide (Liu H. et al., 2020), ALK inhibitors (ceritinib and lorlatinib) (Zhang et al., 2018), autophagy inhibitors (chloroquine and bafilomycin A1) (Torp et al., 2019), and 1,25(OH)2D3 (Cao et al., 2020), have also shown significant inhibitory effects on various aspects of the NEK7-NLRP3 interaction. To better understand the therapeutic effect of molecular inhibitors and medicines, more tightly controlled samples from different species urgently need to be included in future studies.

## Conclusion and Perspectives

In summary, the biological functions of NEK7, including its pathological and physiological effects, have been explored extensively with various approaches from different organisms in the past 20 years. As explained above, NEK7 is mainly involved in various aspects of mitosis regulation and the activation of the NLRP3 inflammasome. The atypical expression and modification of NEK7 has been found to cause cellular oncogenicity and excessive inflammatory responses, thereby leading to the tumorigenesis of multiple organs and aggravating systemic inflammation. This review also summarizes the current inhibitors and medicines targeting NEK7-mediated diseases. However, the regulation mechanism and the effects of the treatment are neither consistent nor assured. Although some reported inhibitors have shown inhibition of NEK7-mediated inflammasome activation, no published works have shown screening of highly selective and potent inhibitors of NEK7. The latest research showed that use of conventional methods led to failed designs of NEK7 inhibitors owing to its unique conformation and high similarity to its paralog NEK6 (Byrne et al., 2020). Then, this group developed a novel strategy using SRS mutations of *NEK7*, which is a promising starting point for developing potent inhibitors targeting NEK7 (Byrne et al., 2020). However, the kinase activity inhibition of NEK7 might have no effect on NLRP3 inflammasome activation (Shi et al., 2016). Therefore, further studies unraveling detailed mechanism and spatiotemporal relationships and searches for more specific inhibitors will provide additional beneficial insights useful for developing more-effective and specific approaches to understanding and treating related diseases.

## Author Contributions

ZS drafted the manuscript. ZJ, YZ, and WG revised the manuscript. All authors contributed to the article and approved the submitted version.

## Conflict of Interest

The authors declare that the research was conducted in the absence of any commercial or financial relationships that could be construed as a potential conflict of interest.

## Abbreviations

NLRP3: NLR Family Pyrin Domain Containing 3
ORF: Open reading frame
Plk1: Polo Like Kinase 1
CDK1: Cyclin Dependent Kinase 1
RGS2: Regulator Of G Protein Signaling 2
EML4: EMAP Like 4
ATP: adenosine triphosphate
GSTO1-1: Glutathione S-Transferase Omega 1
CYLD: CYLD Lysine 63 Deubiquitinase
MTOC: microtubule-organizing center
HDAC6: dynein adapter histone deacetylase 6
LTP: Hippocampal long term potentiation
TRF1: Telomeric Repeat Binding Factor 1
WHSC1L1: Wolf-Hirschhorn Syndrome Candidate 1-Like 2
EML4: EMAP Like 4
FoxM1: Forkhead Box M1
TNM: Tumor Node Metastasis
DSS: Dextran Sulfate Sodium
SRS: Strong R-spine.
